# *Caenorhabditis elegans* as a Convenient Animal Model for Microbiome Studies

**DOI:** 10.3390/ijms25126670

**Published:** 2024-06-18

**Authors:** Cheng-Yeu Wu, Scott Davis, Neekita Saudagar, Shrey Shah, William Zhao, Arnold Stern, Jan Martel, David Ojcius, Hung-Chi Yang

**Affiliations:** 1 Center for Molecular and Clinical Immunology, Chang Gung University, Taoyuan 33302, Taiwan; cyrano@mail.cgu.edu.tw (C.-Y.W.); jan_martel@hotmail.com (J.M.); 2 Department of Endodontics, Arthur Dugoni School of Dentistry, University of the Pacific, San Francisco, CA 94103, USA; sdavis4@pacific.edu; 3 Doctor of Dental Surgery Program, Arthur Dugoni School of Dentistry, University of the Pacific, San Francisco, CA 94103, USA; n_saudagar@u.pacific.edu (N.S.); s_shah19@u.pacific.edu (S.S.); w_zhao3@u.pacific.edu (W.Z.); 4 Grossman School of Medicine, New York University, New York, NY 10016, USA; arnold.stern@nyulangone.org; 5 Department of Biomedical Sciences, Arthur Dugoni School of Dentistry, University of the Pacific, San Francisco, CA 94103, USA; 6 Department of Medical Laboratory Science and Biotechnology, Yuanpei University of Medical Technology, Hsinchu 30041, Taiwan

**Keywords:** *Caenorhabditis elegans*, microbiota, microbiome, host–microbe interaction, microbial metabolite, drug

## Abstract

Microbes constitute the most prevalent life form on Earth, yet their remarkable diversity remains mostly unrecognized. Microbial diversity in vertebrate models presents a significant challenge for investigating host–microbiome interactions. The model organism *Caenorhabditis elegans* has many advantages for delineating the effects of host genetics on microbial composition. In the wild, the *C. elegans* gut contains various microbial species, while in the laboratory it is usually a host for a single bacterial species. There is a potential host–microbe interaction between microbial metabolites, drugs, and *C. elegans* phenotypes. This mini-review aims to summarize the current understanding regarding the microbiome in *C. elegans*. Examples using *C. elegans* to study host–microbe–metabolite interactions are discussed.

## 1. Introduction

### 1.1. C. elegans as a Model Organism

*Caenorhabditis elegans* is a free-living nematode found worldwide in diverse environments, and it usually hosts a microbial community similar to that found in the surrounding environment. The wild-type strain of *C. elegans*—often known as “soil nematode”, Sydney Brenner’s *C. elegans*, or the iconic N2 strain—was isolated from decomposed mushrooms in Bristol, United Kingdom [1]. *C. elegans*, although usually mistaken for a soil nematode, can readily be isolated from rotting vegetables or human-made compost heaps, which have an abundant pool of the nematode’s bacterial nutrition source.

A four-year survey of French orchards by Félix and Duveauin showed the presence of flourishing populations of *C. elegans* in decomposing fruits and plants [2]. *C. elegans* is found in rotting fruits of many kinds, as well as in the rotting stems of herbaceous plants. It is often found with *C. briggsae*. Both species can be found in the same fruit (20% of decaying apples from Orsay, France), and exhibit reproducible seasonal shifts in abundance. Rotting fruits and stems frequently incorporate hundreds of worms and all their life phases, including a rare type of male.

In its natural habitat, the *C. elegans* microbiota includes a gut microbial community and possibly microbes physically associated with its surface. *C. elegans* interacts with a wide range of microorganisms, comprising bacteria, fungi, and other microbes. Various factors, including soil type, geographic location, and environmental conditions, influence the composition of its microbiome [3]. As a result, the microbiome of *C. elegans* may exhibit considerable variation.

### 1.2. Advantages in Biomedical and Microbiome Studies

Symbiotic microbes develop different relationships with their host. Mammalian microorganisms mainly display mutualism in evolution. Disruptions in the balance of the host–microbiome relationship, referred to as dysbiosis, can alter the growth, fitness, and metabolism of the host [4]. This can result in development of various diseases [5]. With an enhanced appreciation of their contributions to host pathophysiology, the exploration of the human microbiota has garnered much attention in recent years [6]. A complete understanding of the complex host–microbiome interactions is essential for developing effective microbial therapeutics. However, it can be challenging to investigate host–microbiome interactions in vertebrate models, as the relationship between an individual member of the mammalian microbiome and the host is influenced by variable factors, such as host genetics, the environment, and heterogeneity of the mammalian immune system.

Studies with other commonly used animals, such as murine models, are limited by the cost and restrictions of high-throughput analysis. The genome of *C. elegans* has been fully sequenced and annotated, and nematodes are highly amenable to genetic manipulations through traditional forward/reverse genetic screening and novel genome-editing technology, such as CRISPR [7]. This organism’s short lifespan is also useful for studying biological phenomena such as longevity.

**Figure 1:**
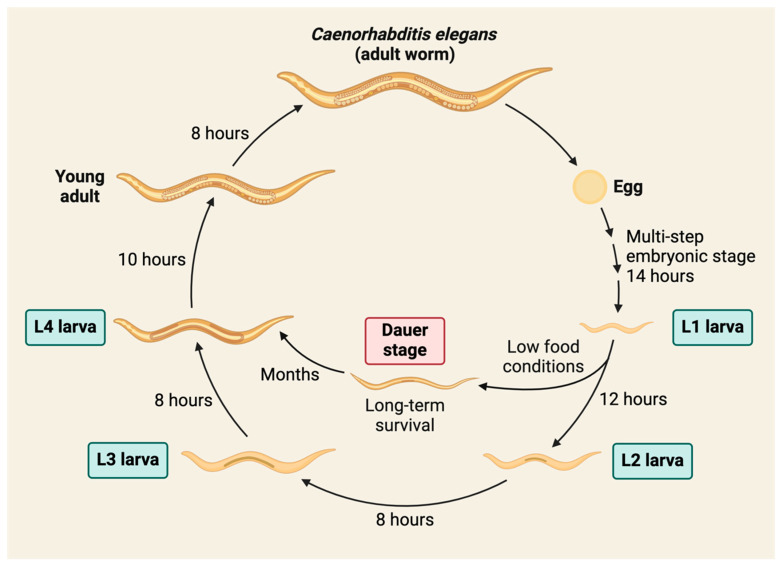
Life cycle of *C. elegans*. Nematodes of the N2 strain can complete one full generation every 3.5 days at 20 °C. The main larval stages (L1 to L4) are indicated, along with the long-term survival stage (dauer), which is induced when food is low or unavailable.

The life cycle of *C. elegans* consists of an initial embryonic stage followed by four distinctive larval stages (L1–L4), which culminate in the adult stage (Figure 1). The N2 strain of *C. elegans* completes one generation every 3.5 days at 20 °C. This organism may enter a long-term survival stage, referred to as *dauer* (which means “duration” in German), as an alternative to the standard L3 larval stage (Figure 1). The distinct developmental stages allow for the study of embryogenesis, development, and dauer formation. The life cycle also allows for the establishment of large-scale cultures for high-throughput study. The transparency of the *C. elegans* body allows direct observations of anatomical structures and fluorescently tagged molecules of interest. Additionally, the nematodes can be easily cryopreserved, and many strains are available from the Caenorhabditis Genetics Center “https://cgc.umn.edu (accessed on 14 June 2024)”. These attributes make *C. elegans* a widely accepted model organism for biomedical research.

*C. elegans* has additional advantages over other models for studying cause–effect relationships between the microbiota and the host. *C. elegans* allows for direct visualization of microbe colonization within the organism through microscopy. In particular, a range of well-established life history readouts pertinent to the effects of *C. elegans*–microbe interactions, such as those related to stress resistance, chemotaxis, foraging behavior, population growth, and fecundity, enhance its utility as a model organism for microbiome research.

## 2. The Microbial World of *C. elegans*

### 2.1. Life in the Wild

In nature, *C. elegans* feeds on various bacterial species found in rotting organic matter, such as rotting fruits, flowers, and plant stems. In its natural environment, *C. elegans* is in constant contact with many other organisms, including other small invertebrates and arthropods, mollusks, bacteria, fungi, and other nematodes. *C. elegans* travels between locations via vectors such as isopods and snails. Nematodes are also prey to a variety of insects and fungi. Fungi can attack *C. elegans* by using trapping structures, such as constricting rings from the hyphal branch of *Drechslerella doedycoides* [8,9]. Other potential predators include mites, springtails, and other nematodes [10]. In the wild, the *C. elegans* gut can accommodate different microbial species. In contrast, it is typically host to a single bacterial species, for example, *Escherichia coli*, in a laboratory setting. The composition of the gut microbe can also vary during the lifespan of the worms.

### 2.2. The Primary and Secondary Microbiomes of C. elegans

**Figure 2:**
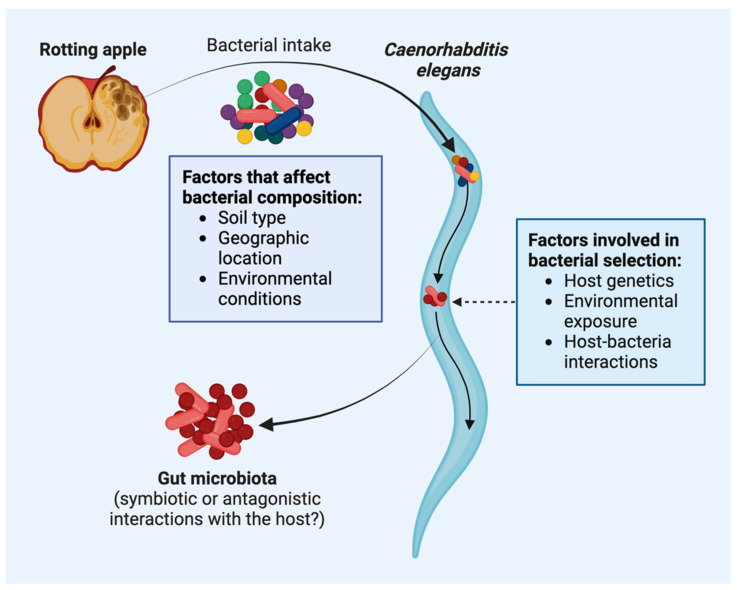
Factors that affect bacterial colonization and host–microbiota interactions in *C. elegans*. In their natural habitat, *C. elegans* nematodes feed on bacteria found on decaying fruits, plants, and organic matter. Various factors affect bacterial colonization and selection in the gut of *C. elegans*, which represents an interesting model organism to study host–microbiota interactions.

In its natural habitat, *C. elegans* shows dynamic interactions with various microorganisms. These interactions can manifest in two distinct forms: mutualistic and antagonistic (Figure 2). Certain bacterial strains form a mutualistic association with *C. elegans*, conferring benefits to them, whereas others may represent a threat or compete for resources.

*C. elegans* is a common inhabitant of temperate regions. It usually lives in decomposing plant material like compost, rotting fruits, and soil, where it interacts with a diverse community of bacteria [10,11]. These bacteria contribute to the development of a microbiome for nematodes. Until recently, the native microbiome of *C. elegans* remained mostly unknown. In 2016, the first three studies to investigate the microbiome of *C. elegans* were conducted [12,13,14]. Utilizing deep sequencing of the 16S rDNA V4 region, a comprehensive analysis revealed the bacterial composition of isolated *C. elegans* from diverse natural habitats across various sampling locations (northern Germany, Portugal, France, and Spain). Operational taxonomic units (OTUs, representing bacterial taxonomic groups) demonstrated that *C. elegans* is associated with a species-rich microbiome, and the bacterial composition is influenced by the host developmental stage and genotype [13]. These microbiomes contribute to the worm’s defense against fungal pathogens [13] and increase lifespan [12]. However, pathogens (such as Bacteroidetes) are associated with the formation of non-proliferating dauers [12]. The findings emphasize the significance of the native microbiome in relation to *C. elegans*.

A reconstructed microbiome, like the native microbiome, can promote growth and reproduction [14]. A comparison of the worm microbiome from diverse sources of bacteria has revealed that the worm microbiome resembles that of *C. elegans*, indicating a shared core gut microbiota [14]. The microbial composition of both native and reconstructed microbiomes has been analyzed through meta-analysis, which revealed that the microbial community composition remains consistent across various locations, study approaches, laboratories, and perturbations during worm processing. This suggests that *C. elegans* actively selects a defined and non-random microbiome from its environment (Figure 2). It remains unclear whether or not this characteristic microbiome is actively selected by *C. elegans* or if the differences in nematode colonization efficacy of the various bacteria play a role [15].

A simplified natural microbiome for *C. elegans*, known as CeMbio, has been developed [16]. It has been derived from a previous meta-analysis of the *C. elegans* primary natural microbiome. This reconstructed microbiome complements the strength of the *C. elegans* model and is a valuable approach for understanding the cause-and-effect relationships in host–microbiome interactions. The variability in microbiome composition within natural populations of worms and their respective environments over two consecutive years has been analyzed by the use of 16S rDNA amplicon sequencing. The development of the native microbiome in *C. elegans* is influenced by factors such as time, habitat (substrate), and the presence of specific bacterial taxa in individual organisms [17]. The impact of long-term fertilization on the *C. elegans* microbiome suggests that its microbiome composition is driven by environmental and host conditions [18].

The development and changes in the microbiome and microbial colonization in a host organism are categorized into two distinct phases—primary and secondary microbiota. Primary microbiota refers to the initial microbiota that establish themselves in the host during the early stages of life, usually acquired during birth or shortly after. As the host organism matures, the secondary microbiota comes into play, involving additional microbial colonization that occurs during later stages of development, influenced by external factors such as environmental exposure and dietary changes (Figure 2). The secondary microbiota further contributes to the complexity and diversity of the host’s microbial ecosystem.

## 3. Experimental Approaches for Studying the *C. elegans* Microbiome

In its journey from nature to the bench, the Bristol (N2) strain has been used in many laboratories and has become the canonical wild-type strain. In the wild, *C. elegans* is predominantly limited to moderate geographical regions [19], while living conditions of *C. elegans* in the laboratory have been modified to adapt to various experimental goals.

In the laboratory, researchers can control environmental conditions, such as temperature, food availability, and light cycles, to a greater extent than in the wild, which allows for a more controlled environment for studying the nematode. In the wild, *C. elegans* is exposed to a wide range of environmental conditions and stresses, which can significantly impact its development, behavior, and survival. Therefore, comparing *C. elegans* in both settings is crucial for comprehensively understanding the nematode’s biology and behavior.

When animals are transferred from their natural habitats to the laboratory environment, they face strong selective pressures that can change them over time. Living conditions are controlled, breeding is regulated, and food is always available. This laboratory environment can significantly impact the animals and alter them genetically due to selective pressures on generations of breeding. Investigators impose novel pressures through culturing, such as transferring individual organisms to start a new culture. The substrate on which the organisms are grown should also be considered. Agar plates offer a two-dimensional substrate, whereas rotting fruit is a three-dimensional environment [11]. Laboratory propagation results in evolution through artificial selection, which inevitably affects model organisms’ genotypic and phenotypic characteristics.

*C. elegans* can be maintained in axenic cultures, which are microbe-free, or cultured under monoxenic cultures, which contain a single bacterial strain. Axenic cultures are pure and do not contain any other organisms that can be used as a food source. These cultures can be either solid (like nutrient agar) or liquid. They contain chemicals or organic material extracts, such as liver. Monoxenic cultures are composed of one organism as a food source. *C. elegans* cultures commonly contain *E. coli* and are either in liquid or solid. Liquid cultures involve growing *C. elegans* in a solution with agitation, while solid cultures involve growing on nematode growth medium (NGM) agar plates having been seeded with *E. coli*. Axenic cultures enhance metabolic activities and are heat-tolerant [20]. They also promote lifespan extension, possibly caused by the effect of dietary restriction [21], since these phenotypes can be reversed by feeding with metabolically active *E. coli*.

*C. elegans* is usually grown on an *E. coli* food source at a temperature of 20 °C. One can also vary the temperature between 15 °C and 25 °C or feed the worms with different bacteria, although it is not clear how these variations affect the worm’s gene expression. The global gene expression of the *C. elegans* N2 strain grown on two different bacterial diets, *E. coli* or *Bacillus subtilis*, has been investigated at 15 °C, 20 °C, and 25 °C [22], and has shown significant metabolic and defense responses occurring in *C. elegans* due to fluctuating the temperatures within the physiological range. The differential pathogenicity between *E. coli* and *B. subtilis* in the diet further alters the worm’s transcriptome, possibly due to the sporulation and nitric oxide formation in *B. subtilis* [23].

The *C. elegans* gut microbiome is mainly composed of fast-growing Gram-negative bacteria. These bacteria are also found in rotting fruits and compete with the host for various types of metabolic interactions. Microorganisms found in compost from natural-like environments have a similar composition of microorganisms to *C. elegans*, which consists of specific microbiota that can be studied experimentally [15].

*C. elegans* has been used to screen and study the effects of gut microbiota [24]. Because of genetic similarities between mammals and the worm, *C. elegans*’ reaction to different environments can resemble its effect on mammals. The approach for conducting experiments using *C. elegans* is seeing how different microbiomes in the human body are metabolized. This is done by testing the worms in different bacterial environments, such as the extracted gut microbes from animal feces, or the standard *E. coli* OP50 diet. Testing *C. elegans* with different gut microbiota or *E. coli* OP50 shows differences in growth and lifespan. *C. elegans* fed on murine gut microbes show reduction in diet preference, body size, fecundity, as well as carbohydrate and lipid content, yet these worms exhibit longer lifespans, similar to the influence of diet restriction in mammals [25]. *C. elegans* is also used for studying human infection with pathogens [26,27] and for screening drug–microbe interactions [28]. Drugs can be tested using different concentrations for determining how they affect the organism while keeping nutrient levels constant [29]. An automated drug screening platform [30] and high-throughput microfluidics [31] have been used for drug discovery.

## 4. Insights into Host–Microbiome Interactions in *C. elegans*

From 2007 to 2016, the Human Microbiome Project (HMP) characterized microbial communities at various sites of the human body, including the skin, gastrointestinal and urinary tracts, and nasal and oral cavities. The HMP findings reveal the abundance, composition, and the potential role of the microbiome in both healthy and unhealthy subjects. These studies indicate that changes in the microbiome are associated with diseases. The HMP expands many research avenues, such as using model organisms, for understanding the interactions between the microbiota and its animal host [32]. The development of simpler models is necessary, as it is challenging to interrogate the causal role of the microbiome shaped by human genetics. This is less known within the HMP due to humans’ varying ethnicities and limited genetic diversity [3].

The current knowledge of exploiting *C. elegans* genetics to study host–microbiome interactions is only the tip of the iceberg, since most studies have explored its interaction with the *E. coli* OP50 strain. The establishment of standard microbiota can decipher the effects of the microbiome on physiological aspects of *C. elegans* [14,16]. While the microbiota colonized in the *C. elegans* gut contains less taxa compared to humans, this feature facilitates complete analysis on simpler microbial communities and results in a detailed understanding of the molecular actions in the host–microbiome interactions. Over 83% of the homology of the proteome is similar to its human counterpart [33]; therefore, the impact of the microbiome on *C. elegans* can shed light on the host–microbiome interactions relevant to the animal and human hosts. However, it is imperative to validate the experimental results from the studies of *C. elegans* in mammalian systems.

Host genetic features play a pivotal role in shaping microbiome assembly within the animal gut. Host intrinsic factors, such as immunity and metabolism, can affect microbial composition. In return, metabolites derived from the gut microbe can modulate the lifespan, fitness, and neural behavior of *C. elegans*. Investigation of these genes linked to specific components of the microbiome can provide insight into the mechanism underlying the host–microbiome interactions (Table 1).

### 4.1. The Protective Role of Microbiome in the Host Immune Response

*C. elegans* coexists with a variety of microbiomes that can greatly influence the host’s fitness, longevity, and survival. A large positive boost in *C. elegans* fitness is observed specifically in proteobacteria-rich environments [13] after initial exposure followed by subsequent exposure to pathogens [34]. *C. elegans* raised in a *Pseudomonas mendocina* gut isolate in the laboratory are partially protected from death and colonization by *Pseudomonas aeruginosa*, but in soil conditions, it has been shown that this exposure completely prevents colonization and death. The mechanism of this relationship is due to enhanced resistance via p38 signaling [34]. This is also seen in fungal exposure, but with a difference between initial and constant exposure resistance. When *C. elegans* is exposed to *Drechmeria coniospora* concurrently with a *Pseudomonas* MYb11 isolate, death is not observed, yet a positive impact on fitness occurs after initial exposure to MYb11 [35]. Combining *Bacteriodetes* and *C. elegans* results in death and growth inhibition [35]. These results reveal the sheer vastness of not only diversity but also interactions with *C. elegans* and its microbiome.

The microbiome of *C. elegans* has a distinct variation from its environment, but this variation is reproducible within a *C. elegans* lineage [36]. Immunity-related genes affect the abundance, function, and stability of the microbiome in *C. elegans* [37,38]. Natural genetic variation in wild strains of *C. elegans* contributes to the assembly of distinct microbiomes [39]. By using a mixed bacterial community mimicking the gut microbiome found in wild *C. elegans* isolates, it was shown that they can exploit several signaling pathways that favor the composition of a distinct microbiome. Altered activity of these pathways through RNAi or mutants correlates with enrichment for specific bacteria; for example, the status of the DAF-2/IGFR insulin signaling pathway plays a critical role in the host selection of commensals, including *Ochrobactrum*. The presence of *Ochrobactrum* in *C. elegans* is associated with increased growth and body size during development [39]. These findings establish a causal relationship between the DAF-2/IGFR signaling pathway and the composition of the *C. elegans* gut microbiome.

### 4.2. The Metabolic Role of the Microbiome in Host Fitness

Fermentation-based metabolism derived from a microbial diet is essential for extending the *C. elegans* lifespan [40]. Similar to humans, *C. elegans* cannot synthesize vitamin B12 [41]. To support growth, metabolism and foraging behaviors, *C. elegans* obtains vitamin B12 from *E. coli*-derived folate. In contrast, folate overload shortens lifespan, while inhibiting folate synthesis increases lifespan in *C. elegans* [42]. Although folate is essential for cellular biosynthesis, folate deficiency does not hinder *E. coli* proliferation, nor does it affect development and fertility in *C. elegans*. Bacterial folate serves as an exogenous signal, specifically stimulating germ cell proliferation in *C. elegans* [43]. This innocuous attribute of bacterial folate suggests its potential as a target for retarding the aging process in the host.

A genome-wide screen of 3792 strains of *E. coli* mutants identified 44 strains that modulate longevity in *C. elegans* through the bacterial metabolite methylglyoxal [44]. These methylglyoxal-deficient mutants activate DAF-16/FOXO signaling, TORC2/SGK-1, and the mitochondrial unfolded protein response of the host. Methylglyoxal promotes senescence in human dermal fibroblasts, indicating the conserved function of methylglyoxal across species. Hence, methylglyoxal derived from the gut microbes can be a new therapeutic target for managing aging-related pathologies.

A healthy microbiome is required for *C. elegans* fitness and lifespan extension [45]. Colonizing members of the native microbiome by *Chryseobacterium* sp. CHNTR56 MYb120 and *Comamonas* sp. 12022 confers resistance to chemical oxidative stress in *C. elegans*, as indicated by progeny output. Combination of both bacterial isolates synergistically enhances lifespan. RNAseq analysis indicates that the enrichment of detoxification pathways, including glutathione, drug and xenobiotic metabolism, are associated with increased expression of cysteine synthase. Nanopore sequencing reveals that the de novo synthesis pathway of vitamin B6 is dominant in both isolates. Supplementation of vitamin B6 with *E. coli* OP50 promoting longevity suggests a potential therapeutic outcome derived from the microbiome, which benefits overall host fitness and longevity.

The anti-diabetic drug metformin provides many benefits in human health, including anti-hyperglycemic and anti-aging effects, at least in part by altering the gut microbiome [46]. Long-term use of metformin in diabetic patients has been linked with vitamin B12 deficiency and peripheral neuropathy [47]. Such phenomena are reversible by administration of antibiotics, indicating that bacteria mediate the effect of metformin-triggered vitamin B12 deficiency [48]. High-throughput analysis of *C. elegans* and multiple *E. coli* strains demonstrates that the transcription factor RcdA is responsible for metformin-induced vitamin B12 accumulation in bacteria. In particular, metformin increases expression of the bacterial B12 transporter in an RcdA-dependent manner, thereby causing vitamin B12 deficiency in the host [48]. Whether metformin can induce a similar effect in other members of the microbiome may be relevant for clinical applications.

### 4.3. The Modulatory Role of the Microbiome in Host Neural Functions

Neuro-gastroenterology is a burgeoning interdisciplinary field dedicated to the study of human health and disease. It revolves around the concept of the brain–gut axis, which denotes the bidirectional communication system connecting the central nervous system (CNS) and the gastrointestinal tract. The gut microbiota regulates the brain–gut axis, while the brain communicates with the intestinal microbiota though several pathways. The cross talk in the brain–gut axis is involved in the development of neurodegenerative diseases. Hence, the gut microbiome provides a target that could be developed for new therapeutic intervention [49].

Bioactive neurotransmitters produced by pathogenic bacteria alter nervous system activity and host behavior, such as olfactory behavior [50]. However, whether the commensal gut microbiome also affects host behavior is unclear. *C. elegans* cultivated on commensal *Providencia* species displays preferential selection of these bacteria in food choice experiments [51]. *Providencia*-derived tyramine exerts neuromodulatory activity on sensory behaviors to promote fitness, such as octanol avoidance, which bypasses the host’s tyramine-producing pathway.

The endogenous neuroinflammatory toxin quinolinic acid (QA) is associated with impaired cognition and neural architecture in diet-induced obese mice [52,53]. QA also disrupts neural signaling and reduces brain-derived neurotrophic factor [54]. In *C. elegans*, QA induces damage in dopaminergic and glutamatergic neurons. Supplementation with butyrate prevents QA-induced neuronal damage and cognitive dysfunction, such as long-term learning and memory decline in *C. elegans* [55]. *Clostridium butyricum*, one of the butyrate-producing bacteria, can ameliorate cognitive decline in mice [56]. This suggests that butyrate-producing bacteria may serve as a probiotic in preventing cognition reduction.

### 4.4. The Protective Role of the Microbiome in Host Protein Homeostasis

Colonization of the *C. elegans* intestine with human gut-associated bacteria disrupts proteostasis [57]. The pathogenic genera, including *Escherichia*, *Klebsiella*, *Proteus*, *Shigella*, *Salmonella*, *Pseudomonas* and *Acinetobacter*, induce protein aggregation in the intestine and in distal tissues, including gonads, muscles, and neurons. The short-chain fatty acid butyrate is produced from dietary fibers during bacterial fermentation [58]. Co-colonization with pathogen and engineered butyrate-producing *E. coli* in the *C. elegans* intestine reduces pathogen-induced protein aggregation. That endogenous butyrate derived from butyrogenic bacteria suppresses protein misfolding and maintains host proteostasis suggests that utilization of butyrogenic bacteria may represent an effective approach for prevention and treatment of neurodegenerative diseases.

**Table 1:** Summary of the host–microbiome interactions.

*C. elegans*	Microbiota	Drug/Chemical	Interaction	Reference
N2	Gut microbiota (MCB) extracted from murine feces	-	Fitness benefits	[25]
N2	Vancomycin-resistant *Enterococcus faecium*	-	Pathogenesis	[26]
N2	*Klebsiella pneumoniae*	-	Pathogenesis	[27]
N2	*E. coli* OP50	Metformin	Therapeutic effects	[28]
N2	*E. coli* OP50 and HT115	Doxycycline	Systemic drug testing	[29]
N2	*E. coli* OP50	Serotonin 5-HT	Systemic drug screening	[30]
N2	*E. coli* OP50	Test Drugs	Systemic drug screening	[31]
N2	Natural-like microbiota isolated from soil	-	Infection resistant	[34]
N2	Natural-like microbiota isolated from soil	-	Gut microbial homeostasis	[14]
N2 and innate immune mutants	Native intestinal microbiota	-	Gut microbial homeostasis	[38]
Wild isolated *C. elegans*	Core natural microbiome (63-member BIGbiome)	-	Gut microbial homeostasis	[39]
N2	*E. coli* OP50	-	Fitness benefits	[40]
N2	*E. coli* OP50	-	Aging	[42]
N2 Germ Cell	*E. coli* OP50	Bacterial extract of the *E. coli* K-12 strain	Germ cell proliferation	[43]
N2	*E. coli* HT115	-	Fitness benefits	[44]
N2	*Chryseobacterium* sp. CHNTR56MYb120 and *Comamonas* sp. 12022 MYb131	Silicon dioxide nanoparticles	Fitness benefits	[45]
N2	Non-pathogenic and pathogenic *E. coli*	-	Pathogenesis of neurodegenerative diseases	[50]
N2	*Providencia rettgeri* PYb007	-	Pathogenesis of neurodegenerative diseases	[51]
N2	*Enterobacteriaceae* family	-	Pathogenesis of neurodegenerative diseases	[57]
N2	*E. coli* OP50	Butyrate and quinolinic acid	Pathogenesis of neurodegenerative processes	[55]
N2	*E. coli* strains	Metformin	Pharmacology of metformin	[48]
N2	*E. coli* strains	5-fluorouracil (5-FU)	Metabolism of the anti-cancer drug 5-fluorouracil	[59]

## 5. Caveats and Limitations of Using *C. elegans*

*C. elegans* provides a unique opportunity for delineating the effect of host genetics on microbial composition in a controlled fashion. However, the microbiome in laboratory-adapted *C. elegans* is less diverse than in natural habitats [14]. The limited genetic variation in laboratory lines compared to outbred strains in the wild also affects the exploration of an association between host genetics and the microbiome. Nevertheless, the *C. elegans* model can facilitate the identification of genes and pathways that are involved in shaping the microbiome.

The metabolism of drugs can be substantially affected by the intestinal microbiota [59]. One of the challenges is the lack of quantitative analysis of the host versus microbes in a living host–microbiome system. A real-time nuclear magnetic resonance (NMR)-based measurement of metabolism of the anti-cancer drug 5-fluorouracil in *C. elegans* and different human gut bacterial strains allows for the dissection of bacterial gene metabolism by delineating differential drug catabolism in bacteria with different genetic backgrounds and the toxicity of the drug response [60]. This platform can reveal the heterogeneous nature of host–microbiome–drug interactions, which cannot be studied using 16S rRNA sequencing.

The impact of host genetics on the microbiome can be studied by analyzing microbial heritability, investigating the association between a particular gene and a microbiome, and genome-wide techniques to explore the association between genetic variation and the microbiome [61,62]. A notable challenge associated with microbial heritability lies in distinguishing between maternal and host genetic effects [63]. The differentiation between microbes transmitted vertically or selected by genetics can be particularly intricate [64].

There are certain limitations in studying the associations of a gene and the microbiome. Since prior understanding of the candidate gene is required, this strategy restricts the possibility of exploration. The microbiome has complex traits that are supported by a collection of genes. Focus on a single gene narrows the scope of the study and yields limited information on the host–microbiome interaction. Genome-wide techniques, such as a genome-wide association study (GWAS) and linkage analysis, also have some drawbacks. To increase statistical power, a GWAS requires a large sample to compensate for the high level of single-nucleotide polymorphisms and the complexity of the microbial population [65]. Genome-wide quantitative trait locus linkage analysis has identified new genetic modifiers of microbiome traits in inflammatory bowel disease, yet there is a need for further fine-mapping to characterize the specific loci involved [66].

## 6. Future Directions in *C. elegans* Microbiome Research

Research on the microbiome of *C. elegans* has provided valuable insights into host–microbiome interactions and their impact on host physiology. Potential future directions in *C. elegans* microbiome studies include the following. 1. Functional characterization of the microbiome components by identifying specific taxa or consortia that affect host traits or behaviors [67]. 2. Mechanistic dissection of the *C. elegans*–microbiome interactions by addressing host signaling pathways, immune responses, and microbial metabolites [68]. 3. The role of the microbiome in host aging by studying the impact of age-related changes in microbiome composition and function on *C. elegans* health and lifespan [69]. 4. Microbiome-mediated stress responses by analyzing how extrinsic stressors, such as pathogens, toxins, or dietary changes, affect host–microbe interactions [69,70,71]. 5. The therapeutic potential of the microbiome by exploring the manipulations of the *C. elegans* microbiome that could be exploited to prevent or cure host diseases [72]. 6. Technological advancement by establishing new tools and platforms, including computer modeling, genome-editing tools, multi-omics, and high-throughput sequencing and imaging [73,74]. Overall, future research on the *C. elegans* microbiome holds great promise for advancing our understanding of host–microbiome interactions and their relevance to health and disease in nematodes, animals, and humans.
